# The Critical Saturation Magnetization Properties of Nanocrystalline Alloy Under Rectangular Wave Excitation with Adjustable Duty Cycle

**DOI:** 10.3390/ma18040735

**Published:** 2025-02-07

**Authors:** Liang Zou, Sixiao Xin, Zhen Li, Yifan Wang, Zhiyun Han

**Affiliations:** School of Electrical Engineering, Shandong University, Jinan 250061, China; zouliang@sdu.edu.cn (L.Z.); 202420765@mail.sdu.edu.cn (S.X.); 202420753@mail.sdu.edu.cn (Z.L.); 202234744@mail.sdu.edu.cn (Y.W.)

**Keywords:** nanocrystalline alloy, micromagnetics, magnetic moment deflection, hysteresis loss, duty cycle, rectangular wave

## Abstract

High-frequency transformers are subject to excitation with a changing duty cycle during operation. Due to magnetic relaxation, the duty cycle of the rectangular wave affects the magnetization time of nanocrystalline alloy for the core material, which affects whether the transformer can reach the saturation operating point. Based on the micromagnetic theory, a three-dimensional model of the nanocrystalline alloy is established, and rectangular wave excitation with different duty cycle *D* is applied to the micro-model. The influence of *D* on the magnetization process is analyzed in terms of the hysteresis loss *P*_v_ and magnetic moment deflection angular velocity *ω*. The results indicate that when *D* = 0.5, *P*_v_ is the smallest, and when *D* increases or decreases, *P*_v_ increases. Furthermore, *P*_v_ remains the same under the rectangular wave excitation that satisfies the sum of different duty cycles of 1. Regarding *ω*, the smallest value occurs at the rising edge of the excitation when *D* = 0.1, while the largest value occurs when *D* = 0.9. During the falling edge stage, *ω* is smallest when *D* = 0.9 and largest when *D* = 0.1. These results demonstrate that the duty cycle *D* influences the magnetization time of the material. Due to magnetic relaxation, changing the magnetization time determines whether the material can reach saturation magnetization. Therefore, there is a critical state, which is defined as the critical duty cycle *D*_c_. The results show that for *D* < 0.5, the range of *D*_c1_ is between 0.2 and 0.21, and for *D* > 0.5, the range of *D*_c2_ is between 0.8 and 0.81. Increasing the amplitude of the excitation source causes a decrease in *D*_c_, while increasing the frequency causes an increase in *D*_c_.

## 1. Introduction

In modern power conversion and transmission systems, high-frequency transformers play a crucial role. From power electronics to communication technology, efficient high-frequency transformer is needed to achieve energy conversion and transmission. With the continuous development of high-frequency transformer core materials, nanocrystalline alloy, especially iron-based nanocrystalline alloy, has attracted much attention as the core material for high-frequency transformers [1,2,3,4]. The unique properties of the material make it outstanding in high-frequency applications. One of the key attributes is their microstructure, where the grain size is significantly smaller compared to traditional grain core materials, which helps to substantially reduce eddy current loss in the core. Additionally, nanocrystalline alloys exhibit superior permeability, high saturation magnetization, and low hysteresis loss, making them one of the best choices in the field of high-frequency transformer core materials. However, as the operating frequency of a high-frequency transformer increases, the demands on the core material’s performance also rise, and its performance is affected by many factors [5,6,7,8,9].

At present, numerous scholars have studied the preparation and soft magnetic properties of iron-based nanocrystalline alloys. In the [10], the use of flux air induction melting instead of vacuum induction melting to prepare Fe-Si-B-Nb-Cu alloy can not only remove impurities but also avoid the formation of an oxidation layer on the alloy’s surface. Liu, C et al. found that nanocrystalline alloy exhibits high saturation magnetization and low coercivity below 5 A/m [11]. Ki-Chan Kim et al. observed that once the saturation magnetization occurs in the narrow yoke, the excitation current that produces the rotating magnetic field will increase [12]. The authors of [13] revealed that experimental dependences in magnetic-field-induced anisotropy cores follow Rayleigh law formulas in weak magnetic fields, while certain empirical relationships for isotropic materials do not apply. From the microscopic point of view, the influence of nanocrystalline alloy takes into account the internal factors of grains or atomic levels such as large molecular crystal clusters. The authors of [14] estimated the core-size distribution of magnetic nanoparticles from three magnetic properties, and the core-size distributions obtained from these properties showed reasonable agreement with each other. The authors of [15] established a three-dimensional mesoscopic model for nanocrystalline alloys and investigated the effects of crystal volume fraction and grain size on high-frequency magnetic losses. The authors of [16] examined the effects of internal microstructure and high-frequency non-sinusoidal excitation on the magnetic loss of nanocrystalline alloys. In previous studies, based on micromagnetic theory, the magnetization process of nanocrystalline alloys affected by various excitations, such as DC bias, square, triangular, and sine waves, was investigated. It was found that micromagnetism can intuitively describe changes in microstress inside the material.

In practical engineering applications, high-frequency transformers are subjected to a range of complex operating conditions [17]. The excitation voltage often contains non-sinusoidal waves, such as PWM waveforms and square waves. The core loss of a high-frequency transformer is closely related to its excitation voltage and saturation characteristics. Zhao Zhigang et al. used the Jordan loss separation model to develop a magnetic core loss prediction model under PWM wave excitation, finding that the prediction accuracy of this model is higher than that of the traditional Steinmetz formula [18]. Venkatraman extended sine wave analysis to the single rectangular waveform encountered in switching power supply converters and analyzed the differences in eddy current losses caused by rectangular and sinusoidal currents [19]. Liu Huan and other scholars have constructed various excitation loss calculation models for medium-frequency and high-frequency transformers [20].

However, the aforementioned references have investigated the calculation methods for high-frequency transformer core loss at the macroscopic level and analyzed the relationship between the structure of nanocrystalline alloys and high-frequency magnetic loss at the microscopic level. The mechanism by which the excited duty ratio influences the magnetic saturation process of high-frequency transformers is challenging, and the application of nanocrystalline alloys in transformer cores lacks theoretical support. First, compared to power–frequency sine excitation, changes in the switching frequency of the system lead to variations in the duty cycle of the external excitation, which results in changes in the magnetization time, thus affecting the magnetization process of the material. Second, the excitation of microwave fields causes various magnetization relaxation processes in the material, leading to energy loss [21]. Sparks M and Patton C discussed this and developed the magnetization relaxation theory [22,23]. The key variable in magnetization relaxation is the magnetization relaxation time. Rectangular waves with different duty cycles may cause the material to reach different magnetization states, with a specific value at which the nanocrystalline alloy is in the saturated magnetization state. This corresponds to the high-frequency transformer operating in the saturated state. When the magnetization time is below the critical value, the transformer operates in an unsaturated state. If the magnetization time exceeds the critical value, the transformer enters the saturated state, resulting in increased core loss, which is detrimental to the long-term stable operation of the high-frequency transformer. Therefore, it is essential to study the effect of the duty cycle on magnetization relaxation and the critical saturation state of nanocrystalline alloy materials.

In this paper, a three-dimensional mesoscopic model of nanocrystalline alloy under mesoscopic conditions is established. The static characteristic parameters of the model are obtained by applying a static magnetic field, and the magnetic loss of the model is obtained by applying an alternating magnetic field, the accuracy of the model is verified by comparing it with the experimental data. The rectangular wave excitation with different duty cycles is applied, respectively, and the hysteresis loss *P*_v_ and magnetic moment deflection angular velocity *ω* are used as the research parameters to explore the influence mechanism of rectangular wave excitation on the saturation magnetization process of nanocrystalline alloy. When the rectangular wave with an adjustable duty cycle is applied to the material, a specific duty cycle value is identified, which makes the material change from being unable to reach the saturation magnetic induction to being able to reach the saturation magnetic induction. This duty cycle is defined as the critical duty cycle *D*_c_. Finally, the influence of changes in the amplitude and frequency of external excitation on the critical duty cycle is also investigated. The study of the critical duty cycle is more helpful in understanding the working state of the high-frequency transformer and provides the groundwork for the selection of the working point of the high-frequency transformer under different working conditions.

## 2. Establishment and Verification of Model

**Figure 1:**
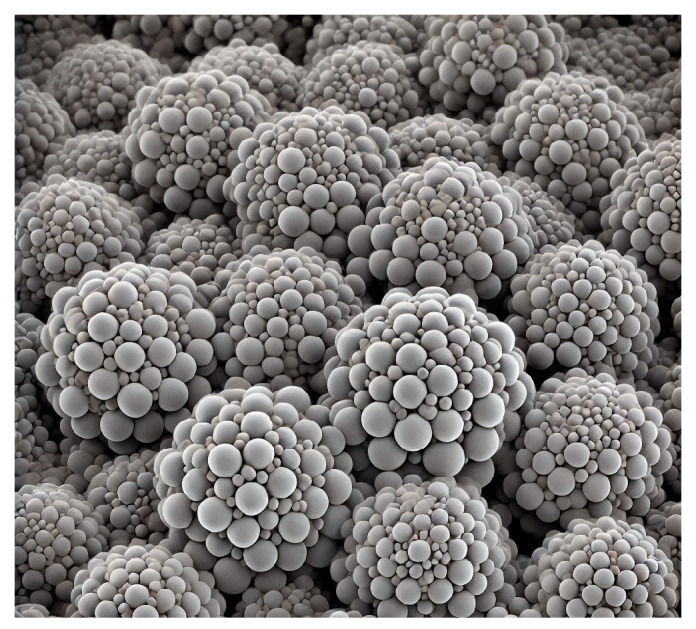
Microstructure of nanocrystalline alloy.

**Figure 2:**
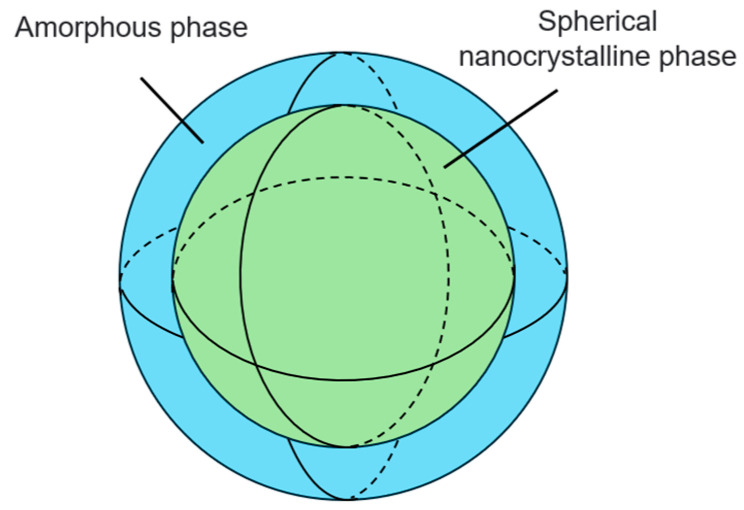
Schematic diagram of spherical nanocrystals.

**Figure 3:**
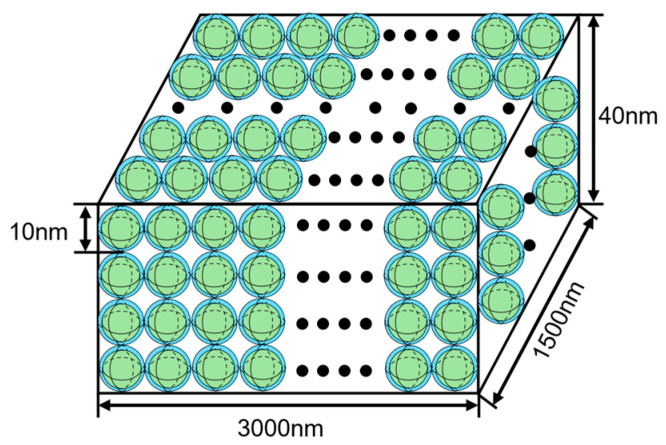
Nanocrystalline alloy model diagram.

Among many types of nanocrystalline alloy, the iron-based nanocrystalline alloy has excellent magnetic permeability and magnetic properties, and the price is lower than other types. Furthermore, FINEMET is selected for modeling. The material is mainly composed of Fe_73.5_Cu_1_Nb_3_Si_13.5_B_9_. The microstructure of the alloy can be observed through the ZEISS-40MAT optical microscope (Oberkochen, Germany), as shown in Figure 1. The microstructure of the nanocrystalline alloy is composed of a nanocrystalline phase and an amorphous phase, the grain size of the spherical nanocrystalline phase is between 10 and 15 mm and dispersed in the amorphous phase, as shown in Figure 2. Based on G. Herzer’s random anisotropy theory, the micromagnetic calculation model of 3000 nm × 1500 nm × 40 nm nanocrystalline alloy is established through micromagnetic simulation software OOMMF 2.0, and the grain size is determined to be 10 nm, as shown in Figure 3.

**Figure 4:**
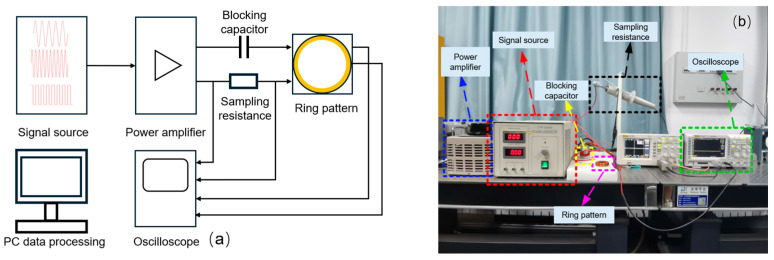
The measurement system for static magnetic properties of the sample. (**a**) Schematic diagram; (**b**) test system diagram.

To verify the accuracy of the simulation model, a system which can test the static magnetic properties is designed based on the magnetic test ring sample of NIM-2000S. The system is shown in Figure 4. The test system is mainly composed of an excitation module, a test module, and a data processing module. The excitation module is composed of a signal source and a power amplifier. The signal source is used to generate an alternating magnetic field with different frequencies and amplitudes, which is amplified by the power amplifier and applied to the primary side of the test sample. The test module consists of a test sample and a straight capacitor in series. The straight capacitor can eliminate the influence of DC bias on the magnetic loss of nanocrystalline alloy. The oscilloscope is used to sample the primary excitation current and the secondary induced voltage of the ring sample, and transmitted to the computer for processing, in which the primary excitation current is obtained by measuring the voltage on the sampling resistance.

During the test, the frequency of the alternating magnetic field is selected as the commonly used application frequency of 10 kHz in nanocrystals. The size of the sample is: inner circle diameter 32 mm, outer circle diameter 50 mm, and thickness 20 mm. Its microscopic parameters are a volume fraction of 60% and a grain size of 10 mm [24].

**Figure 5:**
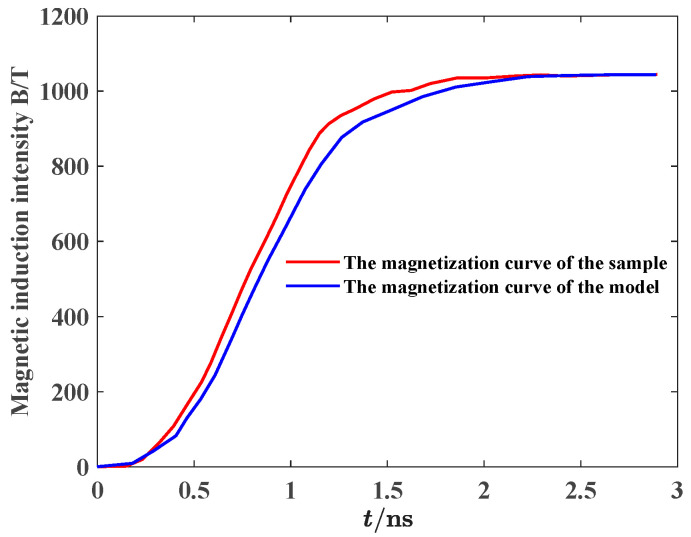
Comparison of magnetization curve between model and experimental sample.

Figure 5 shows the relationship between the magnetic induction intensity and time during the magnetization process of the simulation model and experimental model excited by the same excitation source. The magnetization curve of the nanocrystalline alloy micromagnetic model is not much different from that of the experimental sample. Meanwhile, the static parameters of the simulation model and experimental model can be calculated based on the static hysteresis loops, as shown in Table 1. The static magnetic characteristic parameters of the model have little difference with the experimental data of alloy material, and the maximum error is 2.71%, so it can be considered that the constructed nanocrystalline model is authentic and reliable.

**Figure 6:**
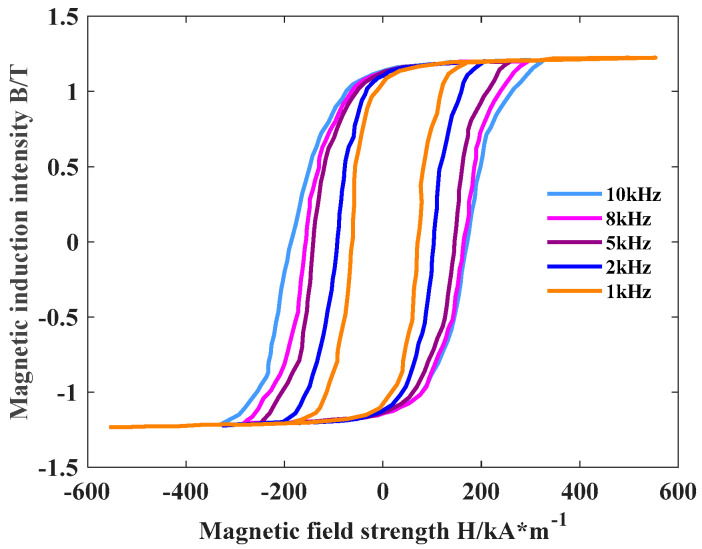
Dynamic hysteresis loops of ring samples at different frequencies.

**Figure 7:**
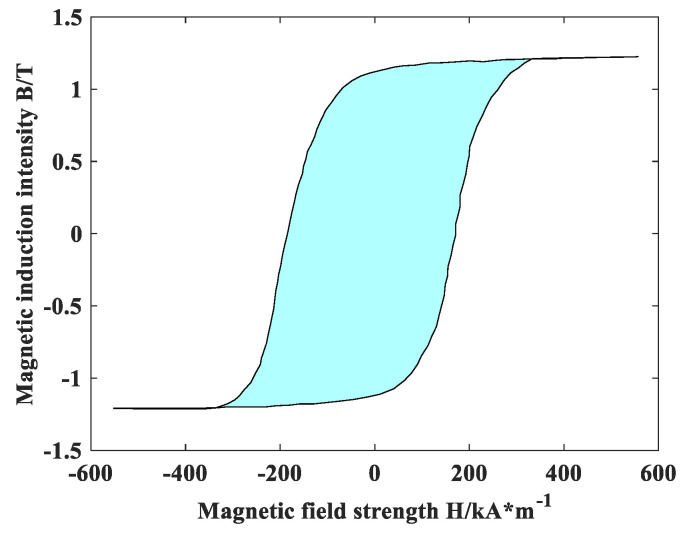
Dynamic hysteresis loop of the model under 10 kHz excitation.

To verify the accuracy of the dynamic magnetic characteristics of the nanocrystalline alloy micromagnetic model, a sinusoidal alternating magnetic field with an amplitude of 0.7 T and a frequency of 1~10 kHz is applied to the toroidal nanocrystalline alloy according to the test system established in Figure 4 to obtain its dynamic hysteresis loop and then obtain the magnetic loss at different frequencies. The dynamic hysteresis loop of the ring under different frequencies and alternating magnetic field excitation is shown in Figure 6. At the same time, a sinusoidal alternating magnetic field with an amplitude of 0.7 T and a frequency of 1~10 kHz is applied to the established micromagnetic model to obtain the dynamic hysteresis loop of the model, and the hysteresis loss of the nanocrystalline alloy is obtained by calculating the enclosed area. Figure 7 shows the dynamic hysteresis loop of the model under 10 kHz excitation.

Table 2 lists the experimental and simulated hysteresis losses under different frequency excitations. There is little difference between the magnetic loss obtained by the model simulation and that measured by the experiment according to Table 2. When the frequency of the alternating magnetic field is 10 kHz, the error is 1.41%. When the frequency of the alternating magnetic field is 1 kHz, the maximum error is 7.45%, but it is still less than 10%, which once again verifies the authenticity of the micromagnetic model of nanocrystalline alloy and also proves that the high-frequency loss of nanocrystalline soft magnetic alloy can be studied by the dynamic hysteresis loop output by the micromagnetic simulation software.

## 3. Influence of Rectangular Wave with Different Duty Cycles on the Magnetization Process

**Figure 8:**
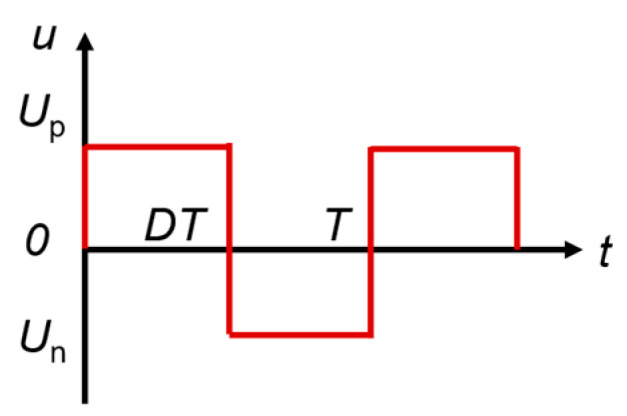
Rectangular wave excitation waveform.

The waveform of rectangular waves is achieved by rapidly switching between high and low levels, forming distinct straight horizontal segments and steep edges. Its waveform changes through four stages: the high-level holding stage, the falling edge stage where the high-level jumps to the low level, the low-level holding stage, and the rising edge stage where the low-level jumps to the high level. The duty cycle *D* is the ratio of the time the high level occupies to the period *T*. The excitation waveform with an adjustable duty cycle is shown in Figure 8, satisfying the mathematical relationship *U*_p_*DT* = −*U*_n_(*T* − *DT*). Where *U*_p_ is the voltage amplitude of the high-level portion of the rectangular wave and *U*_n_ is the voltage amplitude of the low-level portion.

### 3.1. Effect of Duty Cycle on Hysteresis Loss

The different duty cycles can lead to different magnetization times, and changing the magnetization time may cause the high-frequency transformer to operate in oversaturation, resulting in increased loss and increased safety hazards in power grid operation.

**Figure 9:**
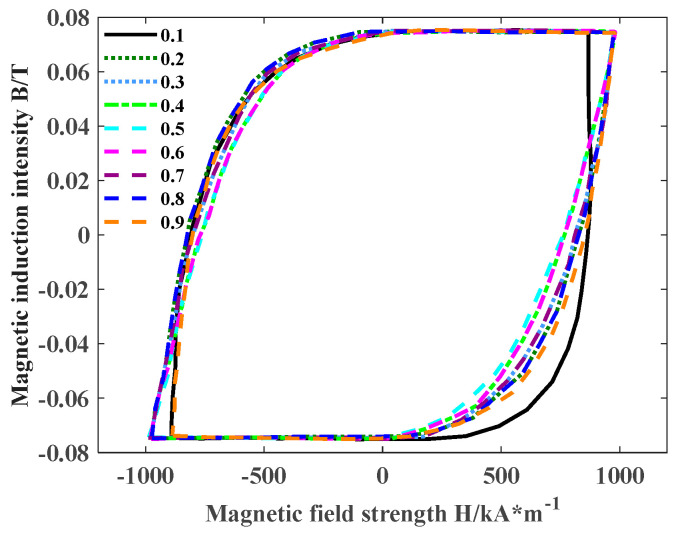
Different hysteresis loops under rectangular wave excitations with different duty cycles.

Apply rectangular wave excitations with a frequency of 10 kHz, amplitude of 25 mT, and different duty cycles to the model. The duty cycle *D* is set to 0.1, 0.2, 0.3, 0.4, 0.5, 0.6, 0.7, 0.8, and 0.9, respectively, and the magnetic hysteresis curves are shown in Figure 9.

It can be observed from Figure 9 that different *D* will lead to different hysteresis loops under rectangular wave excitation. When *D* is 0.4 and 0.6, respectively, the hysteresis loops obtained coincide, which proves that the hysteresis loss generated by nanocrystalline with duty cycles of 0.4 and 0.6 are the same. *D*1 = 0.3 and *D*2 = 0.7, *D*1 = 0.2 and *D*2 = 0.8 also satisfy the relationship, which proves that except for duty cycles of 0.1 and 0.9, when the duty cycles *D*1 and *D*2 of two different groups of rectangular waves satisfy *D*1 + *D*2 = 1, the hysteresis loss is also the same.

**Figure 10:**
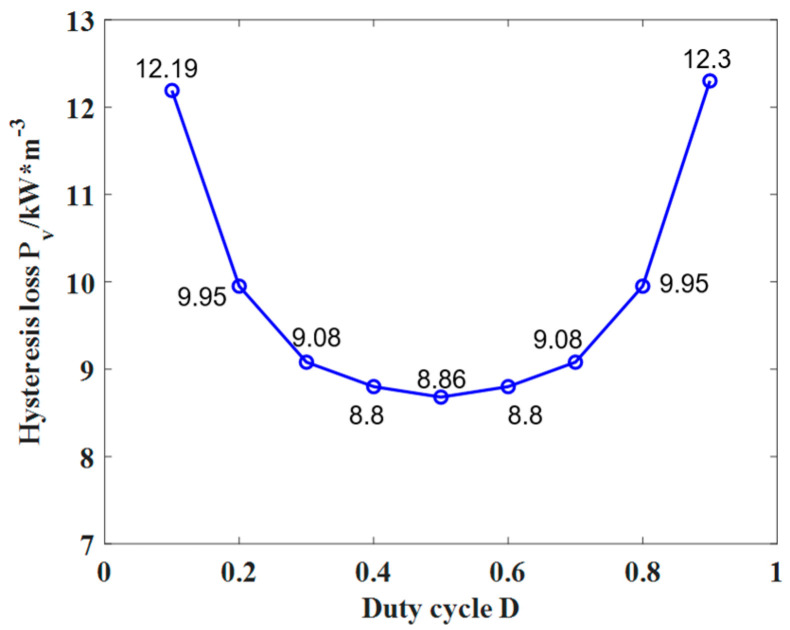
Hysteresis loss *P*_v_ under rectangular wave excitation with different duty cycles.

The hysteresis loss of nanocrystalline under different duty cycles can be calculated by integrating the hysteresis loops of Figure 10.

According to the hysteresis loss under the excitation of rectangular waves with different duty cycles, the distribution relationship of hysteresis loss under different duty cycles can be obtained. It can be seen from Figure 10 that the hysteresis loss under different duty cycles presents the symmetrical distribution of about *D* = 0.5. When duty cycle *D*1 + *D*2 = 1, the distribution of a single duty cycle is asymmetrical, such as *D*1 = 0.4 and *D*2 = 0.6, but the average power and period of the input signal are basically the same, and the amplitude and frequency of the magnetic field changes within each period remain stable, so the hysteresis loss of the transformer remains similar. Comparing the hysteresis loss generated by rectangular waves under different duty cycles, it can be found that when *D* = 0.5, the hysteresis loss generated by the material is the smallest, which is 8.68 kW/m^3^. Whether the duty cycle of the rectangular wave increases or decreases, it can be seen from Figure 10 that the hysteresis loss is gradually increasing. When *D* = 0.9, the hysteresis loss reaches the maximum, which is 12.3 kW/m^3^. When the duty cycle is 0.5, the magnetic field inside the transformer is completely symmetrical; the time, frequency, and amplitude of the magnetization and demagnetization process are uniform; the hysteresis loop area is minimum; and the hysteresis loss is minimum. When the duty cycle deviates from 0.5, the magnetic field changes asymmetrically in the working process of the transformer, resulting in non-uniform energy release, which increases the hysteresis loss. Due to the symmetry, the phenomenon of duty cycles between 0.1 and 0.5 can be studied in a subsequent study.

The most widely used equation for calculating core loss is Steinmetz’s equation [25], but it is suitable for calculating core loss under sinusoidal excitation. Considering that the core loss is related to the magnetic susceptibility, and the magnetic susceptibility is positively correlated with the change rate of magnetic flux density, the equivalent frequency related to the change rate of magnetic flux density is used instead of the original empirical formula to form the modified Steinmetz’s equation, which can be used to calculate the core loss under non-sinusoidal excitation. The equation is as follows [26]:

(1) $$P_{v}=\left(Kf_{\mathrm{eq}}^{\alpha -1}B^{\beta }\right)f$$

where *K*, *α*, *β* is the parameter in Steinmetz’s empirical equation under sinusoidal excitation; *f* is the frequency of the non-sinusoidal excitation waveform; and the equivalent sinusoidal magnetization frequency *f*_eq_ is related to the change rate of magnetic flux density [26]:

(2) $$f_{\mathrm{eq}}=\frac{2}{\Delta B^{2}\pi^{2}}\int_{0}^{T}{\left(\frac{dB}{dt}\right)}^{2}dt$$

In the equation, ΔB is the difference between the maximum and minimum magnetic flux density during a magnetization period. For rectangular waves with different duty cycles, according to the calculation process of the above correction method, the calculation math model of the corresponding core loss can be derived.

Since the rising and falling phases of the rectangular wave are very short and can be approximated as 0, the rectangular wave with different duty cycles is

(3) $$u(t)=\left\{\begin{array}{c} U_{1},0\leq t\leq DT\\ U_{2},DT\leq t\leq T \end{array}\right.$$

ΔB is:

(4) $$\Delta B=2B_{m}=\frac{1}{C}\left(\int_{0}^{\frac{DT}{2}}udt+\int_{\frac{DT}{2}}^{\frac{T}{2}}udt\right)=\frac{U_{1}D+U_{2}\left(1-D\right)}{2Cf}$$

(5) $$\int_{0}^{Τ}{\left(\frac{dB}{dt}\right)}^{2}dt=\int_{0}^{Τ}{\left(\frac{u}{C}\right)}^{2}dt={\left(\frac{U_{1}}{C}\right)}^{2}\cdot \frac{DT}{\left(1-D\right)}$$

where *B*_m_ is the magnetic induction intensity amplitude and *C* is constant.

Substituting Formulas (4) and (5) into Formula (2), we can calculate the following:

(6) $$f_{\mathrm{eq}}=\frac{2}{\pi^{2}D(1-D)}$$

The correction equation of rectangular waves with different duty cycles is

(7) $$P_{v}={\left(\frac{2}{\pi^{2}D(1-D)}\right)}^{\alpha -1}K\cdot f^{\alpha }\cdot B_{m}^{\beta }$$

According to Formula (7), when the two sets of rectangular wave excitation satisfy *D*1 + *D*2 = 1, the hysteresis loss generated by the material is the same.

### 3.2. Effect of Duty Cycle on the Angular Velocity of Magnetic Moment Deflection

**Figure 11:**
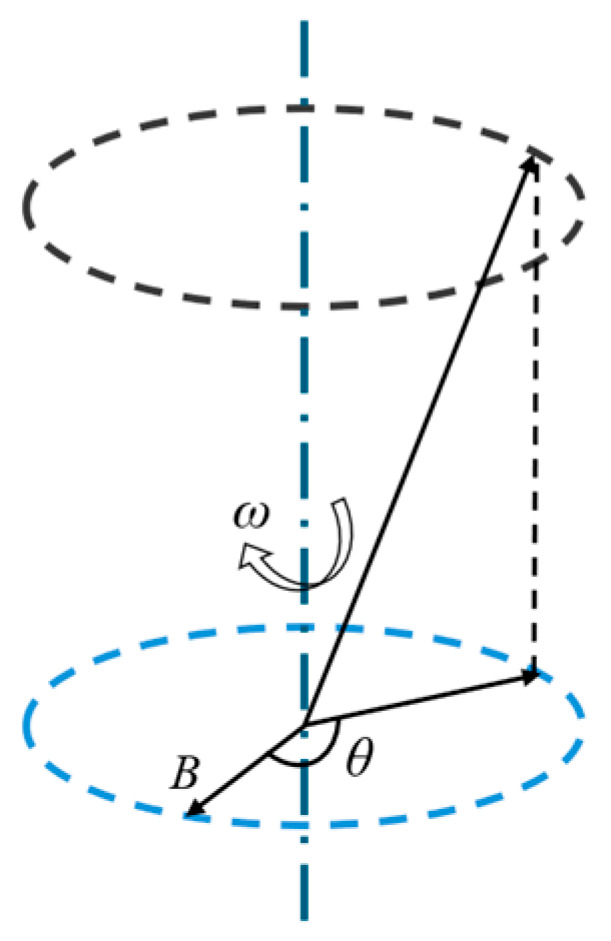
Schematic diagram of angular velocity of magnetic moment deflection.

From the above research results, it can be found that the main stages in which the rectangular wave duty cycle has an impact on the magnetization process of the material are the falling edge of the high-level transition to the low level and the rising edge of the low-level transition to the high level. To describe the speed of the material’s magnetization process at a microscopic level, it can be characterized by the angular velocity *ω* of the magnetic moment deflection, ω=dθ/dt, and the unit of *ω* is rad/ns. *θ* is the angle between the unit magnetic moment and the applied magnetic field, and the unit is rad. Figure 11 shows the schematic diagram of angular velocity of magnetic moment deflection.

**Figure 12:**
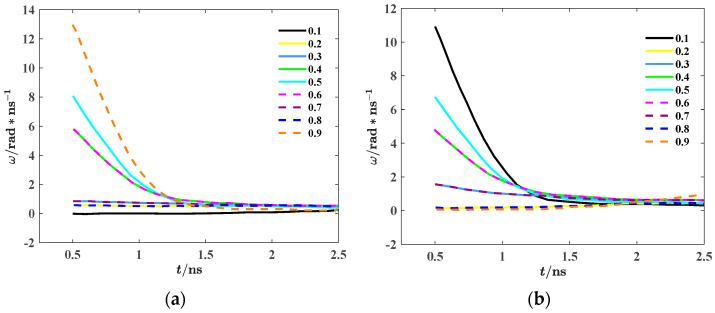
Different ω of rectangular waves with different duty cycles in different stages. (**a**) Rise phase; (**b**) fall phase.

The effect of different duty cycles on the microscopic magnetization process of the material can be observed by studying *ω*. The relationship between the internal magnetic moment deflection angular velocity *ω* and time in two different stages is shown in Figure 12.

It can be seen from Figure 12a that when the duty cycle changes from a low level to a high level, *ω* reaches the maximum at the duty cycle of 0.9 and the minimum at the duty cycle of 0.1. When the duty cycle of the rectangular wave is 0.9, the system stays at a high level for a long time and the low level for a short time. The magnetic moment in the system does not reach complete reverse saturation at the low level, so part of the magnetic moment is still close to the high-level magnetization direction. When the external field switches from the low level to the high level, the magnetic moment that is not completely flipped rapidly responds to the change in magnetic field direction, and the magnetic domain wall moves faster. As a result, *ω* reaches its maximum. At the same time, when the duty cycle is 0.9, because the magnetic domain is saturated in the high-level field, the adjustment time after switching to the high level is very short, and *ω* is significantly increased. Excluding the cases of a duty cycle of 0.1 and 0.9, the angular velocity of magnetic moment deflection under two sets of rectangular waves with different duty cycles but when the sum of duty cycle is 1 is the same. When the duty cycle is 0.1, the angular velocity of magnetic moment deflection is the minimum. This is because when the duty cycle is 0.1, the system is maintained for a long time in the low-level state, the long-term low level field action makes the magnetic moment stabilize and almost completely deflect to the low-level direction, and the system is close to the low-level saturation state. When the external field switches to the high level, the magnetic moment has fully adapted to the low-level state, and more time is needed to overcome the stability of the low-level direction. This delay results in a decrease in *ω*. At the same time, the long time magnetization at low level reduces the activity of the domain walls. When switching to the high level, the initial movement of the domain walls is slow, which reduces the angular speed of the system in response to the external field.

It can be seen from Figure 12b that when the duty cycle changes from the high level to the low level, *ω* reaches the maximum at the duty cycle of 0.1 and the minimum at the duty cycle of 0.9. When the duty cycle of the rectangular wave is 0.1, the duration of the high level is shorter, the external magnetic field can not make the system reach the low-energy stable state (that is, the saturated magnetization state), part of the magnetic moment still maintains the original low level direction, and the magnetic domain arrangement in the system is still in the unstable state of higher energy. When the external field suddenly changes from a high level to a low level, the unsaturated magnetic moment will rapidly respond to the change in the low-level field, and the unstable magnetic domain wall will experience rapid movement to reallocate the direction of the magnetic domain, resulting in *ω* reaching its maximum. At the same time, the system will rapidly release energy, thus showing a higher magnetic moment deflection angle velocity. When the duty cycle is 0.9, the high level lasts for a long time, the external magnetic field has completely deflected most of the magnetic moments to the high-level direction, and the system has reached a state of saturated magnetization. In the saturated state, the magnetic domains have been stably arranged, and the motion of the magnetic domain walls in the system almost stops. When the external field is converted from a high level to a low level, the saturated magnetic moment takes longer to overcome inertia and reorient, so the *ω* is smaller.

## 4. Critical Duty Cycle

Since the hysteresis loss of the material under the influence of different duty cycles has the property of symmetry along *D* = 0.5, this proves that we can reflect the overall properties by studying the properties of *D* in the range of 0.1~0.5. In special cases, that is, when the duty cycle is 0.1, we find that when the rectangular wave is in the critical state of transition from the high level to the low level, the magnetic domain direction does not deflect to the same direction, which proves that the material does not reach its saturation magnetization at the moment before the rectangular wave transition stage, but as the rectangular wave changes to the low level, the material also changes from positive magnetization to negative magnetization. When the duty cycle increases to 0.3, the internal magnetic domain has been deflected to the unified direction, which proves that when the duty cycle is 0.3, the material can reach the saturation magnetization at the moment before the jump stage.

The above phenomenon shows that when the duty cycle is between 0.1 and 0.3, when the rectangular wave is about to jump from the high level to the low level, there is a specific duty cycle, which makes the material change from unable to reach the saturation magnetization to be able to reach the saturation magnetization. Therefore, this specific duty cycle value is defined as a critical duty cycle. Studying the critical duty cycle can more effectively help the high-frequency transformer to be in the most efficient working state, which is helpful to further understand the magnetic behavior of the material.

In the OOMMF software, the step size is 0.01, and the duty cycle is 0.1~0.3. The data are stored in mmDataTable and then screened by Python script to screen out the magnetization intensity of the high-level state that is about to change to the low-level state and compared with the saturation magnetization that the material can achieve.

Table 3 shows the relationship between different duty cycles and the difference between magnetization and saturation magnetization before the jump stage, the specific difference is $\Delta M=M_{m}-M_{s}$, *M_s_* is the saturation magnetization, and *M_m_* is the maximum magnetization of the material in the unsaturation. It can be seen from Table 3 that when the duty cycle is 0.2, the difference between the magnetization in the transition stage and the saturation magnetization that the material can reach is 0.001, and when the duty cycle is 0.21, the difference suddenly drops to 6.79 × 10^−5^ A/m. At this time, the difference between the two has become very small. It can be approximated that the difference is 0, that is, the material has reached saturation magnetization. Therefore, when the difference between the magnetization and the saturation magnetization at the moment before the jump stage is less than 10^−5^ A/m, it can be approximately considered that the saturation magnetization has been reached, that is, the material has reached the saturation magnetization.

When the amplitude is 25 mT, 0 < *D* < 0.5, the value of the critical duty cycle *D*_c1_ should be between 0.2 and 0.21. When 0.5 < *D* < 1, the value of the critical duty cycle *D*_c2_ should be between 0.8 and 0.81. When *D* < *D*_c1_, the material can not reach saturation in the high-level holding stage. When *D*_c1_ < *D* < *D*_c2_, the material can reach the saturation magnetization in both the high-level and low-level stages. When *D* > *D*_c2_, the material can not reach the saturation in the low-level jump stage.

Before reaching the saturation magnetization, it will undergo the period of magnetization relaxation, that is, the magnetic moment gradually changes from the initial state to the direction consistent with the external magnetic field. The time used in this process is defined as the magnetization relaxation time, which is represented by *τ*. The *τ* means that the magnetic moment aligns to the external magnetic field faster, which is smaller, while the larger means that the process of alignment is slower. The relationship between the magnetization intensity of the material and magnetization time can be described by the classic relaxation model, as shown in Formula (8):

(8) $$M(t)=M_{s}\left(1-e^{-\frac{t}{\tau }}\right)$$

where *M*(*t*) represents the magnetization intensity at *t*, where *t* stands for magnetization time, and *τ* denotes the magnetization relaxation time constant. This model can describe the evolution of magnetic moment over time until it reaches an equilibrium state, during which the magnetization intensity *M*(*t*) of the material gradually approaches the value of saturation magnetization *M_s_*.

When the applied magnetic field is excited by the rectangular wave with different duty cycles, resulting in transitions between high and low voltage, the relationship between the magnetization intensity and time is described by Formula (9).

(9) $$M(t)=\left\{\begin{array}{c} M_{s}\left(1-e^{-\frac{t}{\tau }}\right),0\leq t\leq DT\\ -M_{s}\left(1-e^{-\frac{t-DT}{\tau }}\right),DT\leq t\leq T \end{array}\right.$$

The critical duty cycle *D*_c1_ and *D*_c2_ mentioned previously will have an impact on the magnetization process of the material, and it can be observed from Formula (9) that *D*_c1_ and *D*_c2_ affect the relationship between *DT* and *τ*. When *D* < *D*_c1_, that is, *DT* < *τ*, the magnetic moment cannot fully align with the direction of the external magnetic field when the rectangular wave is in high voltage state. Part of the magnetic moment still has a non-zero angle with the external magnetic field, so *M*(*t*) cannot ultimately reach *M_s_*. When *D*_c1_ < *D* < *D*_c2_, that is, *DT* < *τ* < *T* − *DT*, the magnetic moment can deflect to align with the direction of the external magnetic field, regardless of whether it is in the high-level or low-level state, thereby enabling the material to attain saturation magnetization. When *D* > *D*_c2_, that is, *T* − *DT* < *τ*, it is proved that the magnetic moment cannot deflect to align with the direction of the external magnetic field during the low voltage state, which means that *M(t)* will not ultimately reach *M_s_* at this time.

**Figure 13:**
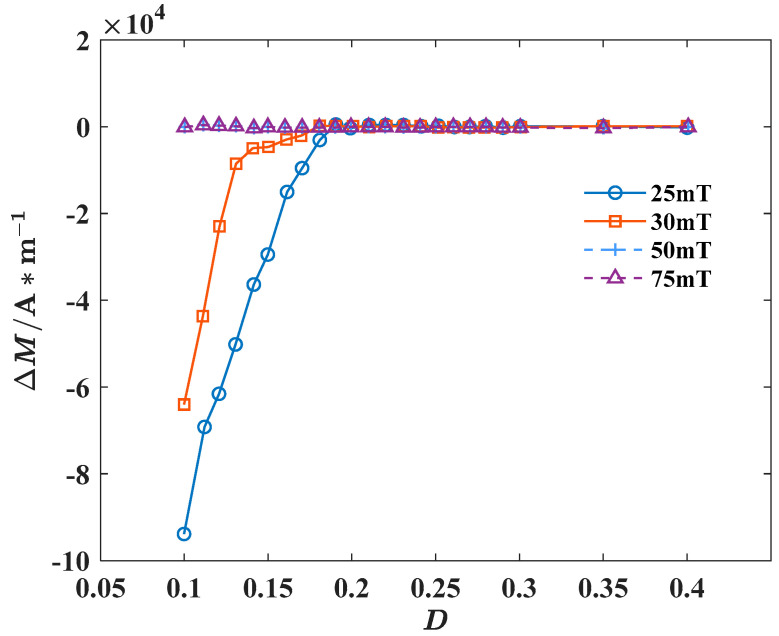
The relationship between Δ*M* and *D* under different amplitudes.

In practical operating conditions, nanocrystalline alloys often encounter varying amplitudes and frequencies of external excitations. To investigate the impact of the two variables, rectangular wave excitations with the amplitude of 25 mT, 30 mT, 50 mT, 75 mT, and the frequency of 10 kHz were applied to the model, respectively. Δ*M* under different amplitude states can be calculated, $\Delta M=M_{m}-M_{s}$, and its relationship with duty cycles is shown in Figure 13.

When the amplitude is 25 mT, *D*_c1_ falls within the range of 0.2 to 0.21. Yet, Figure 13 reveals that when the amplitude is set at 5 mT or 75 mT, the difference between the maximum magnetization intensity and the saturation magnetization is minimal, virtually approaching zero. It suggests that the material attains saturation magnetization in these conditions, with *D*_c1_ falling within the range of 0 to 0.1. While, due to the amplitude of the excitation source increasing, the critical duty cycle of the material decreases accordingly. It is because increasing the amplitude of the excitation source is equivalent to directly increasing the effective force acting on the magnetic moment, which accelerates the process of deflecting the magnetic moment to align with the external magnetic field. This reduces the magnetization relaxation time, accelerates the magnetization process of the material, and enables it to reach saturation magnetization faster.

**Figure 14:**
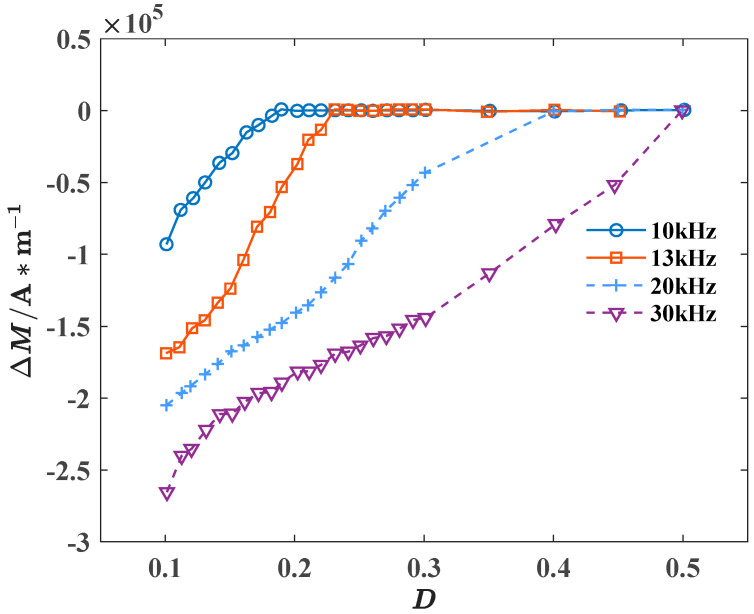
Relationship between Δ*M* and *D* at different frequencies.

On the other hand, when rectangular wave excitations with frequencies of 10 kHz, 13 kHz, 20 kHz, 30 kHz, and the amplitude of 25 mT are applied to the model, respectively, the relationship between Δ*M* and *D* is shown in Figure 14.

Figure 14 reveals that as *D* increases, Δ*M* gradually decreases. While, within the range of *D* from 0.1 to 0.3, only when the frequency is 10 kHz does Δ*M* gradually decrease to 0 as *D* increases, indicating that the material gradually transitions from being unable to reach saturation magnetization to being able to achieve saturation magnetization. When the frequency is 20 kHz, Δ*M* is titchy, which can be approximately regarded as 0. That is to say, the material reaches the saturation magnetization state at this time. *D*_c1_ is between 0 and 0.1, and when the frequency rises from 20 kHz to 30 kHz, Δ*M* gradually increases. It proves that as the frequency of the excitation source increases, Δ*M* also increases, and the critical duty cycle also increases accordingly. This is because increasing the frequency of the excitation source will lead to a decrease in the application cycle of the excitation source, a decrease in the effective time of the external magnetic field acting on the magnetic moment, resulting in a decrease in the effective deflection time of the magnetic moment, and further reducing the magnetization time of the material and slowing down the magnetization process of the material. In summary, the increase in the frequency makes it harder for the material to reach saturation magnetization.

## 5. Conclusions

In this paper, the micromagnetic model is established for nanocrystalline alloy and rectangular wave excitations with the magnetic field of 25 mT and 10 kHz and with *D* of 0.1, 0.2, 0.3, 0.4, 0.5, 0.6, 0.7, 0.8, and 0.9 are applied. The hysteresis loss and magnetic moment deflection angular velocity are analyzed. The conclusions obtained are as follows:

(1) When the duty cycle is 0.5, the hysteresis loss is the smallest. As the duty cycle increases or decreases, it will lead to an increase in hysteresis loss. Except for *D* = 0.5, the hysteresis loss under two sets of rectangular wave excitations with duty cycles summing to 1 is the same.
(2) Under the two stages of rising edge and falling edge, the magnetic moment deflection angular velocity is affected by different duty cycles. When the duty cycle is 0.1, the magnetic moment deflection angular velocity is the lowest during the rising edge, while it is the highest during the falling edge. When the duty cycle is 0.9, magnetic moment deflection angular velocity is the highest during the falling edge, while it is the lowest during the rising edge. For two different groups that satisfy the sum of duty cycles equal to 1, the magnetic moment deflection angular velocity is the same.
(3) The duty cycle of the rectangular wave that can make the material just reach the saturation magnetization is defined as the critical duty cycle *D*_c_. When 0 < *D* < 0.5, the range of *D*_c1_ should be between 0.2 and 0.21. When 0.5 < *D* < 1, the range of *D*_c2_ should be between 0.8 and 0.81. When 0 < *D* < *D*_c1_, it cannot achieve saturation magnetization during the high-level holding stage. When *D*_c1_ < *D* < *D*_c2_, the material can reach the saturation magnetization state during both the high-level and low-level stages. When *D*_c2_ < *D* < 1, it cannot achieve saturation magnetization during the low-level holding stage.
(4) While the frequency and amplitude of the external excitation are altered, the critical duty cycle will also change. On the one hand, when the frequency remains unchanged and the amplitude increases, the increase in Δ*M* will lead to a decrease in the critical duty cycle. On the other hand, when the amplitude remains unchanged and the frequency increases, Δ*M* also increases, leading to an increase in the critical duty cycle.

Critical duty cycle analysis plays an important role in the design and control of high-frequency transformers. In the transformer design, the working point can be determined according to the critical duty cycle to avoid the increase of hysteresis loss caused by over-magnetization. In transformer control, the pulse width modulation strategy can be optimized by analyzing the critical duty ratio so that the transformer can operate in the best magnetization range. The application of the critical duty cycle in the optimization and control of high-frequency transformers can be further studied.

In this paper, the effect of the rectangular wave excitation duty ratio on the high-frequency magnetization process of the nanocrystalline alloy is studied. In practice, temperature has a significant effect on the magnetic properties, such as the magnetic permeability of nanocrystalline alloy materials. The effect of temperature on nanocrystalline alloy materials will be taken into account in the follow-up work, and the effect of temperature on the properties and critical duty cycle of nanocrystalline alloys will be studied.

## Figures and Tables

**Table 1:** Comparison of calculation data and experimental data of nanocrystalline alloy.

	Experimental Data	Calculated Data	Error (%)
Static saturation field strength (kA/m)	225	220	2.22
Saturation magnetic induction (T)	1.24	1.247	0.56
Coercive force (A/m)	310	318.4	2.71

**Table 2:** Comparison between experimental data and simulation data of magnetic loss of nanocrystalline alloy.

	Hysteresis Loss *P*_v_ (kW*m^−3^)				
	1 kHz	2 kHz	5 kHz	8 kHz	10 kHz
Experimental data	7.11	15.27	51.11	100.63	140.01
Calculated Date	7.64	16.35	53.24	102.36	141.98
Error (%)	7.45	7.07	4.17	1.72	1.41

**Table 3:** The difference between the magnetization and the saturation magnetization at the jump stage of rectangular wave excitation with different duty cycles.

*D*	Δ*M*/A*m^−1^
0.1	−107,963.73
0.11	−69,272.39
0.12	−51,538.33
0.13	−23,472.66
0.14	−6141.4
0.15	93.40
0.16	14.96
0.17	0.87
0.18	0.15
0.19	0.008
0.2	0.001
0.21	6.79 × 10^−5^
0.22	1.18 × 10^−5^
0.23	5 × 10^−7^
0.24	8.53 × 10^−8^
0.25	9.55 × 10^−9^
0.26	4.31 × 10^−9^
0.27	3.61 × 10^−9^
0.28	3.61 × 10^−9^

## Data Availability

The data presented in this study are available upon request from the corresponding author.
